# Virtual Alternative to the Oral Examination for Emergency Medicine Residents

**DOI:** 10.5811/westjem.2015.1.24344

**Published:** 2015-02-25

**Authors:** Jillian McGrath, Nicholas Kman, Douglas Danforth, David P. Bahner, Sorabh Khandelwal, Daniel R. Martin, Rollin Nagel, Nicole Verbeck, David P. Way, Richard Nelson

**Affiliations:** * The Ohio State University Wexner Medical Center, Department of Emergency Medicine, Columbus, Ohio; † The Ohio State University Wexner Medical Center, Department of Obstetrics & Gynecology, Columbus, Ohio; ‡ The Ohio State University College of Medicine, Office of Evaluation, Curriculum Research and Development, Columbus, Ohio

## Abstract

**Introduction:**

The oral examination is a traditional method for assessing the developing physician’s medical knowledge, clinical reasoning and interpersonal skills. The typical oral examination is a face-to-face encounter in which examiners quiz examinees on how they would confront a patient case. The advantage of the oral exam is that the examiner can adapt questions to the examinee’s response. The disadvantage is the potential for examiner bias and intimidation. Computer-based virtual simulation technology has been widely used in the gaming industry. We wondered whether virtual simulation could serve as a practical format for delivery of an oral examination. For this project, we compared the attitudes and performance of emergency medicine (EM) residents who took our traditional oral exam to those who took the exam using virtual simulation.

**Methods:**

EM residents (n=35) were randomized to a traditional oral examination format (n=17) or a simulated virtual examination format (n=18) conducted within an immersive learning environment, Second Life (SL). Proctors scored residents using the American Board of Emergency Medicine oral examination assessment instruments, which included execution of critical actions and ratings on eight competency categories (1–8 scale). Study participants were also surveyed about their oral examination experience.

**Results:**

We observed no differences between virtual and traditional groups on critical action scores or scores on eight competency categories. However, we noted moderate effect sizes favoring the Second Life group on the clinical competence score. Examinees from both groups thought that their assessment was realistic, fair, objective, and efficient. Examinees from the virtual group reported a preference for the virtual format and felt that the format was less intimidating.

**Conclusion:**

The virtual simulated oral examination was shown to be a feasible alternative to the traditional oral examination format for assessing EM residents. Virtual environments for oral examinations should continue to be explored, particularly since they offer an inexpensive, more comfortable, yet equally rigorous alternative.

## INTRODUCTION

Simulation-based education and assessment strategies have become increasingly popular in medical education. Healthcare simulations are being used in individual and group settings for both formative and summative assessments.^1^,^2^ The use of immersive learning environments (ILE) for education provides learners with a sense of being immersed in the simulated environment while experiencing it as real. Partial immersive environments involve a virtual world that consists of three dimensions (3-D) displayed on a two-dimensional (2-D) computer screen.^3^,^4^ Educational research using 3-D virtual worlds and their effect on learning outcomes is limited.^5^ With a predicted paradigm shift in medical education where immersive environments continue to expand in the personal and professional lives of learners, it is imperative to explore and understand the implications and limitations of these ILEs.^6^

**Figure 1:**
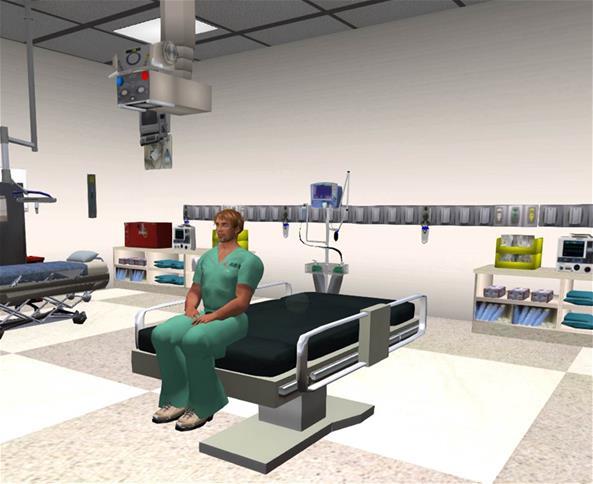
Avatar patient in an emergency department examination bay.

A number of ILEs have been developed in the past ten years with varying rates of adoption by the education community. Second Life (SL) is a virtual 3-D platform that allows individuals from any geographic location to interact in a virtual environment. Accordingly, SL provides opportunities for remote virtual simulation experiences.^5^,^7^ In SL, users are represented in a virtual world by their avatars. An avatar is an online, self-created, animated characterization of the user that can act in any role (doctor, patient, nurse, or teacher) and perform programmed tasks (Figure 1). SL has been successfully used in medical and public health education.^7^,^8^ Specifically, the platform has been used to model doctor-patient relationships, teach clinical diagnosis, train for disasters, virtually tour the human anatomy, and to conduct physical examinations.^4^,^8^–^11^

The American Board of Emergency Medicine (ABEM) administers an oral board examination semiannually to residency-trained emergency medicine (EM) physicians. Passage of the oral board examination is required for EM board certification.^12^ The purpose of the oral board examination is to assess the candidates’ medical knowledge, clinical reasoning, and interpersonal skills. In the current format, candidates travel to a central assessment venue to take the oral board examination. The examination requires the candidate to verbally explain how they would handle various patient cases to an examiner. Many residency programs offer “mock” or practice oral examinations to prepare residents for the ABEM oral boards. Residents at this academic EM residency program participate in an annual mock oral examination, which is conducted in the traditional format, a face-to-face interaction with an examiner. The purpose of this study was to assess the feasibility of using immersive virtual simulation technology to administer oral examinations to EM residents and to evaluate the potential of this platform as an alternative to the traditional face-to-face oral examination.

## METHODS

We used a prospective, stratified-random control group study design to evaluate two methods of administering an oral examination: the traditional face-to-face method and the immersive virtual simulation method. To create a virtual environment we used SL (Second Life 2.0 Viewer, Linden Research, Inc. (Linden Lab), San Francisco, CA). Second Life Viewer is free, open-access computer software; however, fees are required to purchase virtual real estate or to construct virtual environments. We constructed a virtual emergency department (ED) for this study on virtual real estate purchased by one of the authors for another project. Both real estate and building costs were covered by internal institutional grants. The study was conducted at an American, university-based, three-year EM residency training program. Residents at all three levels, program years (PGY) 1–3 were included.

EM residents (n=35) were randomly assigned to one of two groups using a stratified approach to ensure that each group had an equal number of residents from each of three levels of training (PGY1–3). The first group was administered the oral examination using the immersive virtual interface with the examiner at a different physical location than the examinee (Figure 1). The second group served as a control and was administered the oral examination using the traditional format: a face-to-face patient case scenario that was managed with the examiner present in the same room as the examinee. Both groups were administered the same case scenario in which the examinee was expected to diagnose and manage a patient with ST-elevation acute myocardial infarction and resuscitate the patient after cardiac arrest. The study was reviewed and approved by our institution’s behavioral sciences institutional review board.

**Figure 2:**
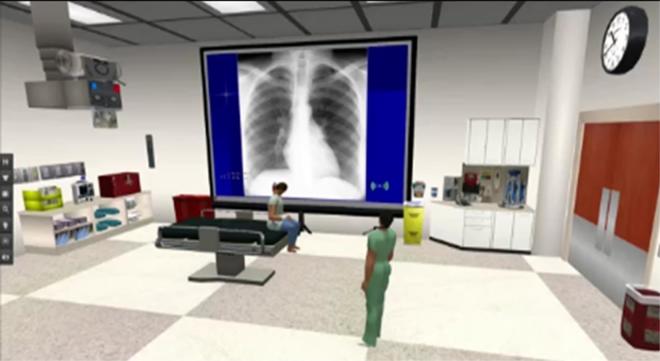
Physician (examinee) avatar examining a patient in an emergency department examination bay after requesting chest radiograph.

In the immersive virtual condition, the examinee managed the patient case using the physician avatar to play the role of the physician (Figure 2). The faculty proctor played the role of the patient using the patient avatar. The examinee and proctor were in remote physical locations and communicated via headset and computer. Resident-examinees verbally interviewed the patient-proctor for historical details and physical exam findings. A collection of pertinent diagnostic data was created in PowerPoint and subsequently loaded into an image viewer in the immersive virtual environment. The faculty proctor controlled the image viewer, allowing diagnostic data (initial and repeat vital signs, laboratory reports, and diagnostic imaging) to be displayed in the virtual examination room in real time when requested by the examinee or at appropriate times during the case (Figure 2). Identical images were printed on paper and offered in similar sequence for the traditional oral exam format. Two faculty proctors administered all virtual examinations and two other faculty proctors administered all traditional oral exams. Access to a video demonstration of the virtual examination can be found at http://vimeo.com/user29472626/videos (Password = OSUEMSL2, case sensitive).

We used the ABEM instruments for scoring resident performance and documenting execution of “critical actions” on both the virtual and traditional oral examination conditions. Using this instrumentation, proctors scored examinees on eight performance items using an 8-point rating scale. The items on the instrument represent the eight ABEM competency categories. Proctors also used the ABEM checklist to document whether the examinee executed 10 “critical actions” during their work on the case. Traditional and virtual groups were compared on: the number of critical actions they executed, their original scores on the eight performance items, and on passage of the performance items as defined by ABEM. The ABEM standard for passing is a composite score greater than or equal to 5.75 on the 8-point scale. For our purposes, all performance items were dichotomized into pass-fail variables to compare groups on pass-rates across each competency category.

Participants were surveyed regarding their opinions about the oral examination experience. The immersive virtual group received a survey comprised of 13 items that used a 5-point Likert response set (from 1=Strongly Disagree to 5= Strongly Agree). Questions inquired about past experiences with the format, any difficulties encountered during the exams, and format preference for the virtual group. Questions further elicited the level of perceived realism, objectivity, efficiency and intimidation during the examinations. The traditional oral examination group received a shorter 6-item survey comprised of questions designed to compare their experience with that of the virtual group.

We used Fisher’s exact tests for analyzing 2 × 2 tables to compare the groups on number of critical actions executed and on pass rates for each of the eight examination items (ABEM competencies and overall clinical competence). Independent t-tests were used to compare the groups on mean item scores for each of the eight items. We used Bonferroni corrections to control for family-wise Type 1 error rates for each set of multiple comparisons (critical action and pass-fail comparisons, and comparisons between competency category scores).^13^

## RESULTS

Fisher’s exact test showed no significant differences between traditional and virtual examinees in the number of critical actions executed (Table 1) and showed no significant difference in pass rates between traditional and virtual examinees (Table 2). We compared groups on examination scores for the ABEM’s individual competency categories and found no significant differences (Table 3). However, moderate effect sizes were observed for many of the ABEM competency categories, with all but two mean differences favoring the virtual examination group (data acquisition and interpersonal relations competencies). The assessment results observed during this study were consistent with results of mock oral examinations administered in prior years.

The mock oral examination is a program requirement for our residency program. Accordingly, all examinees, regardless of training level, had experience with the traditional face-to-face oral examination format prior to this study. Most of the examinees (57%) had never used SL prior to the examination. Examinees reported that both formats were realistic (Traditional 80% vs. Virtual 86%). All examinees perceived the examinations to be fair, objective, and efficient in either format. None of the examinees in the virtual group found the examination to be intimidating (Traditional 40% vs. Virtual 0%), and many reported that the virtual format was less intimidating than traditional oral exams that they had experienced in the past (77%). Most of the virtual examinees reported a preference for the virtual examination (79% agree and 21% neutral) over the traditional format (Table 4).

## DISCUSSION

Criticisms of the traditional oral examination process have been raised by EM residents, including recent residency graduates preparing for the ABEM oral examination.^14^ These criticisms can be classified into three domains or issues; fidelity, validity, and logistics.

The first issue has to do with the oral examination fidelity. While the goal of the ABEM oral examination has been to replicate a realistic ED encounter; the traditional oral examination format, with its face-to-face questioning process, remains somewhat artificial. Also related to fidelity is the manner in which the patient information is communicated to the examinee. Until recently, ABEM examinees have been unable to visualize their patient during the test. Additionally, physical examination findings and diagnostic imaging were presented on paper, rather than in the medium in which it would be encountered in a real ED. To address this issue, ABEM has recently committed to incorporating some computer-based images into their oral examination (eOrals); however, the specific details of this change have yet to be revealed.^12^

A second issue involves the validity of the oral examination format. Threats to validity involve both the effects of performance anxiety and examiner bias. Performance anxiety can occur when an examinee is confronted with an unfamiliar examiner during a face-to-face encounter in an unfamiliar environment. Furthermore, despite formal scoring systems and examiner training, examiner bias also remains a threat to the validity of the traditional oral exam format.^15^ The literature suggests that bias is common towards candidates with good interpersonal skills, good communication skills, and those who are physically attractive.^16^ Finally, a third issue with the traditional ABEM oral examination format involves logistics, such as the expense of preparation and travel to the testing site.^15^

The virtual examination offers many potential benefits to address the issues involved with the traditional oral examination. The virtual examination offers higher fidelity realism than the traditional oral exam by immersing the examinee in a setting resembling one in which actual patient care is delivered. The virtual examination involves dynamic interaction between the examinee as physician and the examiner as patient. Patient information, such as vital signs and diagnostic imaging results, are presented in a more authentic manner in the virtual environment.

Threats to examination validity are also minimized in the virtual examination format. The potential for anxiety produced by a one-on-one encounter with a stranger is alleviated by the virtual world encounter. Examiner biases resulting from the personal encounter with the examinee are also minimized.^3^ Finally, because the virtual examination can be administered through the electronic medium of the world-wide web, logistic concerns can be addressed. Delivering the oral exam through a virtual reality platform eliminates the need for examinees and examiners to travel, providing economic and time savings.

The results of this study offer confirmation that virtual examination results are comparable to those of the traditional oral examinations for assessing EM residents. In fact, we observed moderate effect sizes favoring the SL group, even with relatively small samples, on five of the eight ABEM competencies; suggesting that with bigger samples we might have demonstrated significantly better performance on these competencies through the virtual examination platform.

One can envision the application of advanced virtual simulation technology as a way to alleviate some of the barriers encountered in the current process. In addition to reported ease of use and perception by many that this was a more realistic experience, the virtual examination format is adaptable. The virtual oral examination could be administered from any remote location with computer access, at any time of day. Thus, oral examinations could be completed while on away rotations, while travelling, at home rather than in an office setting, or at a remote testing site.

The implementation of virtual technology in resident assessment required a time commitment for brief training of faculty to use the system to administer the examinations. Direct costs included purchasing space in the virtual world and costs for building the desired assessment environment.^17^ Examiners of the immersive virtual oral examination required 2–3 hours of training in order to develop and monitor the data display. Communication via microphone and computer did not require specific training for the faculty or residents participating in the examinations. Some examinees reported feedback or echoing in the headset. Such impediments can be eliminated through use of higher quality microphones, headsets and computer systems.

Resident feedback regarding the use of the immersive environment for the oral examination in EM was overwhelmingly positive. Many examinees expressed an interest in even more advanced capability to interact with the virtual patient and within the examination room. With currently available animation and programming capabilities, items in the room can be made more interactive. The examinee might click on the patient-avatar’s body to perform physical examination skills or a virtual IV pole to order IV fluids. They might also instruct a nurse avatar to perform programmed tasks. The virtual environment could be expanded to a multi-case format, requiring concurrent care of multiple patients requiring the examinee-physician avatar to transition between patient rooms. More complex immersive assessment environments may offer higher fidelity assessment potential without additional cost or barriers to ease of use. Transition to an automated scenario, without a real-life examiner, could be achieved through application of artificial intelligence. The Unity 3-D platform is an example of another immersive environment that is ideally suited to support the virtual assessment format as it offers higher levels of fidelity and is reported to be easier to use by both examinees and proctors.^18^ In addition, it can be highly customized and configured to provide secure examinations without requiring the type of third party support which is necessary with platforms such as Second Life.

## LIMITATIONS

This study was conducted at a single academic training site and therefore the number of assessment subjects was not large. Our intent was to evaluate the feasibility and value of the virtual examination; however, because each resident experienced only one examination format, they were unable to preference one format over the other. Prior experience with the traditional face-to-face oral examination made it possible for the immersive exam participants to contrast the virtual with the traditional oral exam experience. As voices were not modified in this virtual examination, there is the potential for bias to be introduced based on voice recognition of the examinee or examiner. This is a bias that could be avoided in a larger-scale examination format using anonymous proctors, technology to modify or standardize the proctor’s voice, or creation of an automated case using artificial intelligence. Because only one examiner evaluated each examinee, inter-rater reliability was not evaluated; therefore, inter-rater reliability remains an issue to be studied in future research. Other aspects of testing via virtual simulation require additional exploration before such technology is more broadly adopted for general use in a high-stakes oral board examination. Further research is needed to study faculty perceptions about the virtual examination experience or evaluate the reproducibility of results using this platform.

## CONCLUSION

The virtual simulated oral examination is a feasible alternative to the traditional oral examination format for EM residents. This study used Second Life as a platform for the virtual examination; however, we believe that other immersive learning environments should be evaluated. Future studies should focus on identifying and developing the most user-friendly platforms for virtual oral examination and continue to assess applications of virtual examination in other areas of medical education.

## Figures and Tables

**Table 1:** Frequencies, (percentages), and Fisher’s exact test value for 17 Traditional Oral Exam Group residents and 18 Immersive Virtual Exam Group residents on execution of 10 critical actions during an oral examination case.

		Resident results		
			
Critical action	Group	Missed (%)	Completed (%)	Fisher’s exact test
Check bedside blood glucose	Traditional	10 (58.8)	7 (41.2)	1.00
	Virtual	11 (61.1)	7 (38.9)	
Initiate cardiac monitoring	Traditional	0 (0)	17 (100)	Constant
	Virtual	0 (0)	18 (100)	
Identify inferior wall myocardial infarction	Traditional	0 (0)	17 (100)	Constant
	Virtual	0 (0)	18 (100)	
Administer antiplatelet agent	Traditional	0 (0)	17 (100)	1.00
	Virtual	1 (5.6)	17 (94.4)	
Administer anticoagulation	Traditional	3 (17.6)	14 (82.4)	0.10
	Virtual	0 (0)	18 (100)	
Arrange for emergent cardiac catheterization	Traditional	0 (0)	17 (100)	Constant
	Virtual	0 (0)	18 (100)	
Administer chest compressions/CPR	Traditional	0 (0)	17 (100)	0.49
	Virtual	2 (11.1)	16 (88.9)	
Administer epinephrine or vasopressin	Traditional	0 (0)	17 (100)	0.49
	Virtual	2 (11.1)	16 (88.9)	
Defibrillate pulseless Vtach/Vfib	Traditional	0 (0)	17 (100)	1.00
	Virtual	1 (5.6)	17 (94.4)	
Administer antiarrhythmic medication	Traditional	4 (23.5)	13 (76.5)	0.18
	Virtual	1 (5.6)	17 (94.4)	

*CPR,* cardiopulmonary resuscitation; *Vtach*, ventricular tachycardia; *Vfib*, ventricular fibrillation

* A family-wise Bonferroni correction was used to control for Type I error rates (finding significant differences by chance). The corrected p-value considered for statistical significance is equal to 0.005.

** Critical actions were documented by the proctors using a checklist during the examination.

**Table 2:** Frequencies, (percentages), and Fisher’s exact test value for 17 Traditional Oral Exam Group residents and 18 Immersive Virtual Exam Group residents on passing or failing 8 American Board of Emergency Medicine competency categories.

		Resident pass-fail results		
Competency category	Group	Passed (>5.75) (%)	Failed (<5.75) (%)	Fisher’s exact test
Data acquisition	Traditional	15 (88.2)	2 (11.8)	0.23
	Virtual	12 (66.7)	6 (33.3)	
Problem solving	Traditional	9 (52.9)	8 (47.1)	0.31
	Virtual	13 (72.2)	5 (27.8)	
Patient management	Traditional	9 (52.9)	8 (47.1)	0.74
	Virtual	11 (61.1)	7 (38.9)	
Resource utilization	Traditional	14 (82.4)	3 (17.6)	0.66
	Virtual	16 (88.9)	2 (11.1)	
Health care provided	Traditional	10 (58.8)	7 (41.2)	0.73
	Virtual	12 (66.7)	6 (33.3)	
Interpersonal relations	Traditional	11 (64.7)	6 (35.3)	1.00
	Virtual	11 (61.1)	7 (38.9)	
Comprehension of pathophysiology	Traditional	10 (58.8)	7 (41.2)	0.29
	Virtual	14 (77.8)	4 (22.2)	
Clinical competence	Traditional	10 (58.8)	7 (41.2)	0.29
	Virtual	14 (77.8)	4 (22.2)	

* A family-wise Bonferroni correction was used to control for Type I error rates (finding significant differences by chance). The corrected p-value considered for statistical significance is equal to 0.006.

** A score of 5.75 or greater was required for passing each competency category.

**Table 3:** Means, (standard deviations), independent t-test results, and effect sizes for 17 Traditional Oral Exam Group residents and 18 Immersive Virtual Exam Group residents on 8 American Board of Emergency Medicine competency category scores.

	Group				
				
Competency category	Traditional	Virtual	t	df	Effect size
Data acquisition	6.18 (0.64)	5.94 (1.30)	0.67	25	−0.23
Problem solving	5.59 (0.94)	6.17 (0.92)	1.84	33	0.60
Patient management	5.18 (1.13)	5.89 (1.37)	1.67	33	0.55
Resource utilization	6.06 (0.66)	6.50 (0.86)	1.70	33	0.56
Health care provided	5.53 (1.07)	6.28 (1.07)	2.07	33	0.67
Interpersonal relations	5.76 (0.83)	5.72 (0.83)	0.15	33	−0.05
Comprehension of pathophysiology	5.94 (0.90)	6.28 (1.02)	1.03	33	0.35
Clinical competence	5.59 (1.00)	6.28 (1.02)	2.02	33	0.65

* A family-wise Bonferroni correction was used to control for Type I error rates (finding significant differences by chance). The corrected p-value considered for statistical significance is equal to 0.006.

** Scores were assigned by proctors using a standard ABEM 1–8 scale.

**Table 4:** Means, (standard deviations), independent t-test results, and effect sizes for 10 Traditional Oral Exam Group residents and 14 Immersive Virtual Exam Group residents on six post-examination evaluation items over the interface they experienced: Immersive Environment or Traditional Face-to-Face interface with a proctor.

	Group				
				
Evaluation item	Traditional	Virtual	t	df	Effect size
Comfort: I felt comfortable communicating with the interface (SL or proctor) during the exam	4.60 (0.52)	4.43 (0.65)	0.69	22	0.29
Realism: The interface provided a realistic patient encounter	4.20 (1.03)	4.36 (0.75)	−0.43	22	−0.18
Intimidation: The interface was intimidating	2.60 (1.27)	1.79 (0.58)	1.90	11.7	0.82
Fairness: The interface was fair and objective	4.40 (0.52)	4.43 (0.51)	−0.13	22	−0.06
Efficient: The interface is an efficient way to complete mock oral examinations	4.30 (0.48)	4.29 (0.61)	0.61	22	0.02
Preference: I would prefer to complete more of my oral examination requirements using this interface	3.50 (1.18)	4.29 (0.83)	−1.93	22	−0.77

* A family-wise Bonferroni correction was used to control for Type I error rates (finding significant differences by chance). The corrected p-value considered for statistical significance is equal to 0.006.

** Option key: 1= Strongly Disagree, 2= Disagree, 3= Neutral, 4= Agree, 5= Strongly Agree.
